# Immune-maximizing (IMAX) therapy for cancer: Combination of dendritic cell vaccine and intensity-modulated radiation

**DOI:** 10.3892/mco.2013.108

**Published:** 2013-04-26

**Authors:** YUTA SHIBAMOTO, MASATO OKAMOTO, MASANORI KOBAYASHI, SHIHO AYAKAWA, HIROMITSU IWATA, CHIKAO SUGIE, YOKO MITSUISHI, HIDENORI TAKAHASHI

**Affiliations:** 1 Department of Radiology, Nagoya City University Graduate School of Medical Sciences, Aichi 467-8601;; 2 Institute for Advanced Medical Research, Keio University School of Medicine, Tokyo 160-8582;; 3 Seren Clinic Nagoya, Aichi 460-0008;; 4 Department of Radiology, Nagoya Daini Red Cross Hospital, Aichi 466-8650;; 5 Clinic Saint Louis, Kyoto 607-8011;; 6 Seren Clinic Tokyo, Tokyo 108-0071, Japan

**Keywords:** dendritic-cell vaccine, intensity-modulated radiation therapy, stereotactic body radiotherapy, immune-maximizing cancer therapy

## Abstract

A dendritic cell (DC)-based vaccine was combined with intensity-modulated radiotherapy (IMRT) or other conformal radiotherapy (RT), assuming minimal immunosuppression by such RT modalities. In this study, the outcomes in the first 40 patients are presented. The patients had recurrent, metastatic or locally advanced tumors. Nine had previously undergone full-course RT. Peripheral blood mononuclear cells obtained by leukapheresis were cultured with granulocyte-macrophage colony-stimulating factor, interleukin-4, OK-432 and prostaglandin E2 to generate DCs, which were pulsed with autologous tumor lysates or tumor-specific peptides, such as WT1. IMRT using tomotherapy, stereotactic irradiation or 3-dimensional conformal RT (3DCRT) was initially administered. The standard dose was 30 and 60 Gy in patients with and without previous RT, respectively. Every other week thereafter, up to a total of 7 times, DC vaccines were injected directly into the tumor (n=15) or administered intradermally when DCs were pulsed with tumor lysates or peptides. The tumor response was evaluated according to the response evaluation criteria in solid tumors (RECIST). RT and DC vaccines were well tolerated and there were no major complications. Three patients were not able to complete the planned DC therapy due to disease progression. For the 31 patients receiving full-dose RT, the response rate was 61% and for the 9 patients who had previously received RT, the response rate was 55%. In 9 patients, the tumor response outside the RT target volume was evaluable: 22% had a partial response (PR), 33% had stable disease (SD) and 44% had progressive disease (PD). In conclusion, a combination of IMRT (or 3DCRT) and DC vaccine is feasible and requires further investigation.

## Introduction

Despite recent advances in cancer therapy, several types of advanced tumor remain incurable and the development of new modalities is required. Cancer vaccines, including dendritic-cell (DC) vaccine, are an approach at a crucial crossroads, creating therapeutics designed to target tumor-associated antigens. DCs are professional antigen-presenting cells that play a key role in initiating adaptive and innate immune responses. Since their identification by Steinman (1), attention has been focused on the role of DCs in eliciting an antitumor effect and in potential therapeutic applications and the insight recently gained may provide the basis for generating more effective antitumor immune responses (1-4). With the injection of DCs loaded *ex vivo* with tumor-associated antigens, the DCs induce antigen-specific cytotoxic T lymphocytes (CTLs), which attack cancer cells. Thus far, the DC-based vaccine has had some success, as sipuleucel-T (Dendreon Co., Seattle, WA, USA) was approved as the first anticancer vaccine by the US Food and Drug Administration in 2010 (5). However, cure of advanced cancers is impossible with DC therapy alone, due to its modest efficacy (6-8).

To improve the therapeutic efficacy, therefore, combination with radiotherapy (RT) and chemotherapy is currently under consideration. Previous studies suggested that certain chemotherapeutic agents, including gemcitabine, 5-fluoro-uracil (5-FU) and a metronomic dose of cyclophosphamide, as well as irradiation, may stimulate the host immunity or inhibit suppressors of host responses, such as regulatory T cells (Tregs) and myeloid-derived suppressor cells, enhancing the effect of cancer immunotherapy (9-11). Okamoto *et al* (12,13) reported that a relatively low dose of 5-FU led to the enhancement of anticancer immunity mediated by the induction of helper T-cell type-1 cytokines (such as interferon-γ and interleukin-12) and natural-killer cell activity in patients with oral cancer. They also reported that DC therapy in combination with an oral fluoropyrimidine, S-1, enhanced host immune responses and elicited a marked anticancer effect, as compared to DCs alone in tumor-bearing mice (14), and that DC vaccine combined with gemcitabine and/or S-1 possibly prolonged the survival of patients with advanced pancreatic cancer (15). However, conventional radiation and high-dose chemotherapy generally suppress the immunity of patients; therefore, such combinations may not be the optimal solution (16,17). However, recently-developed intensity-modulated RT (IMRT) focuses radiation beams on the target, minimizing the irradiation of normal tissues; therefore, the immune suppression may be less extensive compared to that in the case of conventional RT. Furthermore, low-intensity chemotherapy, termed tumor dormancy therapy, does not seem to severely affect host immunity and its combination with DC therapy is conceivable. Thus, we recommend an immune-maximizing (IMAX) cancer therapy that combines DC vaccine with IMRT and/or tumor dormancy therapy.

Another rationale for combining DC vaccine with IMRT is that several patients with recurrent cancer have already undergone RT. In such patients, a second course of radiation is often hazardous when a conventional technique is applied. However, re-irradiation is possible with IMRT or similar techniques, since critical organs and normal tissues are mostly spared. Therefore, we attempted to combine DC vaccine and IMRT using tomotherapy or similar conformal RT techniques. When the tumor has an almost spherical shape, 3-dimensional conformal radiotherapy (3DCRT) or stereotactic body radio-therapy (SBRT) is used instead of tomotherapy, since all these radiation modalities yield similar excellent conformal dose distributions.

Regarding treatment with a DC vaccine, our methods have two characteristics. First, whenever possible, the DC vaccine was injected directly into the tumor; using this method, DCs were expected to identify and capture apoptotic tumor cells as antigens *in situ* and induce antigen-specific CTLs thereafter. Second, use of synthetic peptides derived from known tumor-associated antigens, such as Wilms’ tumor protein (WT1) (18) and MUC1 (19), were pulsed into DCs during culture. WT1 and MUC1 reportedly have the first and second highest priorities, respectively, for cancer vaccines among the currently available cancer antigens (20). In this study, the method of IMAX cancer therapy and results from the first 40 patients were reported.

## Materials and methods

### Study design and eligibility

This study aimed to evaluate the feasibility, toxicity and efficacy of combining IMRT, 3DCRT or SBRT and DC vaccine therapy in patients with recurrent or far-advanced malignancy. Eligibility criteria were as follows: i) recurrent or far-advanced cancer considered incurable by standard therapy; ii) World Health Organization performance status of 0–3; iii) expected survival time of ≥3 months; iv) delivery of radiation doses of ≥30 Gy considered feasible by way of IMRT, 3DCRT or SBRT; and v) availability of at least one cancer antigen or feasibility of local DC vaccine injection. Any type and size of malignancy were considered eligible. The study was approved by the respective institutional review boards and written informed consent was obtained from the patients.

### Patients

Between 2007 and 2011, 40 patients were considered eligible and were enrolled in the study. The characteristics of the patients and their diseases are provided in Table I. Twenty-six patients had recurrent malignancies and 9 of these had previously undergone RT. The remaining 14 patients had locally far-advanced tumors.

### DC vaccine therapy

On the first visit to one of our immunotherapy clinics, patients were evaluated on their eligibility for enrollment and availability of cancer antigens. Human leukocyte antigen (HLA) compatibility was assessed for the possible use of cancer antigen peptide(s). Whether the DC vaccine could be administered directly into the tumor was also evaluated. When DC therapy was considered applicable, the attending physicians referred the patients to the Department of Radiotherapy at Nagoya City University Hospital and DC-based immunotherapy was scheduled. During the RT course, DC vaccines were prepared. Our methods for the preparation of DC vaccine were previously described in detail (15).

Briefly, shortly prior to the initiation of radiation, a peripheral blood mononuclear cell (PBMC)-rich fraction was obtained from patients by leukapheresis (400 ml × 13 cycles) using AS-TEC 204 (Fresenius Kabi, Bad Homburg, Germany). The PBMCs were isolated from the heparinized leukapheresis products by Ficoll-Hypaque gradient density centrifugation, as previously described (15) and placed into 100-mm plastic tissue culture plates (Becton Dickinson, Franklin Lakes, NJ, USA) in AIM-V medium (Gibco, Gaithersburg, MD, USA). After 30 min of incubation at 37°C, non-adherent cells were removed and the adherent cells were cultured in AIM-V supplemented with granulocyte-macrophage colony-stimulating factor (500 ng/ml; Primmune, Inc., Kobe, Japan) and interleukin-4 (IL-4; 250 ng/ml; R&D Systems, Inc., Minneapolis, MN, USA) to generate immature DCs (21). The population of adherent cells remaining in the wells consisted of 95.6±3.3% CD14^+^ monocytes (21). After 5 days of culture, these immature DCs were stimulated with OK-432 (10 *μ*g/ml; Chugai Pharmaceutical, Tokyo, Japan) and prostaglandin E2 (50 ng/ml; Daiichi Fine Chemical Co., Ltd., Toyama, Japan) for 24 h. We, as well as other investigators, previously reported that OK-432 matures DCs via Toll-like receptor 4 signaling and that these DCs are effective inducers of antigen-specific CD8^+^ CTLs (14,21–26). It has also been reported that prostaglandin E2 facilitates the ability of DCs to migrate to the lymph nodes (27). DCs generated in this manner were pulsed with 20 *μ*g/ml of peptides for WT1 (18), Her2 (28) and/or CEA (29) at 24 h after the treatment with OK-432 and prostaglandin E2. MUC1 long peptide (30 mer at 20 *μ*g/ml) (30), CA125 protein (500 U/ml) (31) and/or autologous tumor lysates (50 *μ*g/ml) were then added to the DC culture media at the same time as OK-432 and prostaglandin E2 and then incubated for 24 h.

To prepare the autologous tumor lysates, tumor sections obtained by surgical resection were homogenized. The tumor cells were obtained by depleting lymphocytes from the total cell suspension using immunomagnetic bead-conjugated monoclonal antibodies against CD2, CD14 and CD19 (Dynal Biotech, Oslo, Norway) according to the manufacturer’s instructions. Aliquots of the isolated tumor cells were then lysed by placing them through 3 freeze (in liquid nitrogen)-thaw (in 37°C water bath) cycles. The lysed cells were centrifuged at 800 × g for 5 min and the supernatants were passed through a 0.22-*μ*m filter (Millipore Corporation, Bedford, MA, USA). The protein contents of the resultant cell-free lysates were determined using DC protein assay kits (Bio-Rad Laboratories, Hercules, CA, USA). Aliquots (500 *μ*g/tube) were then stored at −135°C until use (32). The DCs were cryopreserved until the day of administration. Surface molecules expressed in the DCs were determined using flow cytometry. The cells defined as mature DCs were CD14^−^, HLA-DR^+^, HLA-ABC^+^, CD80^+^, CD83^+^, CD86^+^, CD40^+^ and CCR7^+^.

Two weeks after the completion of RT, the injection of cultured DCs was initiated, and DC vaccine was administered every other week, up to a total of 7 times. For head and neck cancers, DCs were injected directly into the tumor using a 21-gauge needle; for esophageal cancer, DCs were injected locally through endoscopy; and for liver and lower abdominal bulky tumors, DCs were injected under ultrasound guidance. Otherwise, the DC vaccine was administered intradermally. Thereafter, patients were observed without chemotherapy for a period of at least 3 months. Eight patients had received chemotherapy with S-1 or gemcitabine prior to RT, but chemotherapy was interrupted during the IMAX therapy.

### Radiation therapy

Tomotherapy (Accuray Inc., Sunnyvale, CA, USA) was used to deliver IMRT. Our method of tomotherapy was previously described in detail (33). A 2.5-cm field width was used in the majority of patients. Generally, a pitch of 0.3 and a normal modulation factor of 2.0 were used. The inverse planning was performed with a variable number of iterations, with a range of 50–500 during the optimization process per plan. The patients began treatment with daily mega-voltage CT acquisitions for setup verification. Tomotherapy is useful for irregular-shaped tumors; however, for spherical or almost spherical tumors, 3DCRT and SBRT provide a similar conformal dose distribution. Since several patients were on the waiting list for tomotherapy, spherical or almost-spherical tumors, such as lung metastasis and pancreatic cancer, were treated with 3DCRT or SBRT with 6- or 10-MV X-rays of a linear accelerator using rotational or multiple fixed beams, as previously described (34–36).

IMRT and 3DCRT were administered 5 times a week, with a daily dose of 2 Gy. The total radiation dose was 60 Gy for previously unirradiated patients and 30 Gy in patients who had previously undergone full-dose RT. Changes in the total dose within ±15% were allowed, depending on the patient conditions. For 2 patients with lung or liver metastases, SBRT was performed according to our protocols of 50 Gy in 4 fractions for lung tumors and 55 Gy in 10 fractions for liver tumors (35,36).

### Evaluation of outcome

Patients were followed regularly by physical examination, blood tests and appropriate imaging methods. Seven patients underwent immunological evaluation; the results of which are to be presented elsewhere, including more patients enrolled in other studies (Kobayashi *et al*, unpublished data). Toxicities were initially scored with the Common Terminology Criteria for Adverse Events version 3.0 and then reclassified according to version 4.0. The tumor response was classified as complete response (CR), partial response (PR), stable disease (SD) or progressive disease (PD), according to the response evaluation criteria in solid tumors (RECIST) at the time of maximal tumor regression or at 3 months after the completion of treatment for the evaluation of SD or PD. The survival time of the patients was calculated from the initiation of the RT using the Kaplan-Meier method.

## Results

### Treatment and adverse events

The 40 patients completed the planned RT. DC immunotherapy was then initiated; however, 3 patients were not able to complete the planned courses of DC vaccine administration, due to disease progression. The administered courses of DC vaccine were 1–7 (median, 5). Adverse events possibly related to the DC vaccine were as follows: a grade 2 skin rash on the injected site was observed in 3 out of the 25 patients (12%) and a grade 1 skin reaction was observed in 12 patients (48%). A grade 2 adverse event possibly related to RT was anorexia, observed in 5 patients (13%). No other grade 2 or higher toxicities were observed.

### Tumor response

The responses of the irradiated tumors are summarized in Table II. For the 31 patients receiving full-dose RT, the overall response rate (CR+PR) was 61% and for the 9 patients who had received previous RT, the overall response rate was 55%. In 9 patients, the tumor response outside the RT target volume was evaluable: 22% had PR, 33% had SD and 44% had PD. Thus, the disease control rate (PR+SD) outside the RT target volume was 56%. Of these 9 patients, 4 had received intratumoral DC vaccine and 3 (75%) showed PR or SD, while in the remaining 2 patients exhibiting disease control, the antigens for DCs were a tumor lysate and WT1+MUC1, respectively.

For the 31 patients receiving full-dose RT, the median survival time was 11 months and the 2-year survival rate was 43%. The median survival time was 7.5 months, for the 8 patients with pancreatic cancer and the 6 patients with recurrent head and neck cancer. For the 9 patients who had received previous RT, the median survival was 10.5 months and the 1-year survival rate was 38%.

## Discussion

The combination of RT and immunotherapy has received increasing attention. Although the synergy between these two modalities has been controversial, it has been suggested that RT may activate tumor immunity (37,38). In a recent study, local irradiation induced an increase in the major histocompatibility complex (MHC) or high-mobility group box 1 protein that induces DC maturation (38). Despite these observations, however, host immunity may decrease when the irradiated volume increases. Therefore, we developed the concept of IMAX cancer therapy combining DC vaccine and IMRT or conformal RT.

The intratumoral injection of DC vaccine in combination with RT has been reported in patients with soft tissue sarcoma and prostate cancer (39,40). In those studies, DCs were injected during conventionally fractionated RT. Concurrent use of DC vaccine and radiation may raise concern that DCs subjected to radiation may be functionally impaired. Experiments on mice demonstrated the rapid migration of injected DCs from the tumor site to regional lymph nodes (41). We injected DCs after the completion of conformal RT to avoid this complication. The DC vaccine was used in an adjuvant setting, with intratumorally injected DCs being expected to identify apoptotic tumor cells as antigens *in situ* and deliver information to killer T cells. In the present study, control of distant tumors was observed in 3 out of 4 patients with measurable metastatic lesions who received local irradiation followed by intratumoral DC injection. This rare phenomenon is known as the ‘abscopal effect’. It has been reported that irradiation may induce immunogenic death of cancer cells, enhance antigen presentation by DCs and cause antigen-specific immune responses (38). Furthermore, recent studies demonstrated that RT promotes tumor-specific effector CD8^+^ T cells via DC activation (42) and that γ-irradiation enhances the immunological recognition of cancer cells through the increased expression of cancer/testis and MHC class I antigens (43). These immunological effects of irradiation may induce disease control at distant sites and DC administration may enhance the abscopal effect. The therapeutic methodology for obtaining this effect should be determined. Postow *et al* (44) recently reported that the combination of a monoclonal antibody that inhibits an immunological checkpoint on T cells, CTL-associated antigen 4 and RT induced cancer antigen-specific immune responses and the regression of distant metastases. We hypothesized that a DC vaccine may be useful for achieving this purpose.

The WT1 peptide, alone or in combination with other peptides in the DC vaccine, was most frequently used in the present study. WT1 and MUC1 were ranked as the two most highly immunogenic peptides in a variety of cancer tissues (20). Peptides of the tumor antigen pulsed to DCs were selected according to antigen expression in tumor cells, as well as to the structural HLA restriction of each peptide. In our previous study, however, the frequency of WT1-specific CTLs detected by the WT1 HLA-A*2402 or 0201 tetramer assay did not increase following vaccination in approximately half of the patients (15). The evaluation of immune responses by the tetramer assay yields quantitative but not functional data and is not necessarily correlated with therapeutic efficacy (45). The accumulation of tumor antigen-specific CTLs in the tumor tissue is critical for the evaluation of the antigen-specific immune response. Future studies should be conducted to elucidate these issues by analyzing local tumor microenvironments using immunohistocheminal staining and a DNA microarray system, as well as the clinical usefulness of tumor-specific peptide-pulsed DC vaccine therapy. Furthermore, other immunological responses, including CTLs (CD8^+^) and natural killer cell numbers prior to and following DC vaccine therapy, are currently being monitored. Preliminarily, increases in the CTL and natural killer cell numbers have been observed in a number of patients. In our previous study, the administration of gemcitabine with or without S-1 in combination with DC vaccination tended to lead to a reduction of the Treg number and percentage in the treated patients (15). Patients with a decrease in the Treg number following treatment tended to exhibit longer survival compared to those with an increase in the Treg number. Another study suggested that the reduction of Tregs by gemcitabine possibly contributed to the favorable outcome in the combined treatment of peptide vaccine-based immunotherapy and gemcitabine against advanced pancreatic cancer (46).

Since RT and DC vaccine were administered sequentially, no synergy of adverse events developed and the treatment toxicities were acceptable. There was no development of autoimmune disease. PRs or CRs of the irradiated tumors were observed in approximately two-thirds of the patients. This observation is not surprising considering the efficacy of RT against various tumors. The evaluation of the efficacy of DC vaccine on local tumors was challenging, due to its combination with RT, which has been proven to be effective in the majority of tumors. The overall survival of the patients was generally poor, which was expected, since the majority of the patients had advanced-stage malignancies. Therefore, additional studies are required to evaluate the survival benefit of the DC vaccine.

In conclusion, we recommend IMAX cancer therapy combining a DC-based vaccine and IMRT. This treatment is feasible and has low toxicity. Its clinical efficacy, including survival benefit and immunological responses, is currently being evaluated by ongoing studies.

## Figures and Tables

**Table I:** Patient and treatment characteristics.

Characteristic	Value
Age (years)	
Median (range)	62 (38–84)
Gender	
Male/female	22/18
Primary tumor site	
Head and neck	10
Pancreas	8
Lung	4
Esophagus	3
Uterus	3
Other	12
Radiation modality	
IMRT/3DCRT/SBRT	23/15/2
Radiation dose (Gy), median (range)	
Previous radiation	
− (n=31)	60 (50–62.5)
+ (n=9)	30 (25–30)
Antigen for DC	
Tumor lysate	5
Local tumor	15
Peptide	20
WT1+MUC1/WT1/MUC1+CEA/	6/3/3/2/2/4
WT1+CEA/WT1+CA125/other	
Site of DC administration	
Tumor	15
Intradermal	25
DC course number, median (range)	5 (1–7)

IMRT, intensity-modulated radiotherapy; 3DCRT, 3-dimensional conformal radiotherapy; SBRT, stereotactic body radiotherapy; DC, dendritic cell; CEA, carcinoembryonic antigen; WT, Wilm’s tumor; CA, carbohydrate antigen.

**Table II:** Tumor response.

Group	N	CR	PR	SD	PD	% (CR+PR)
Previously unirradiated	31	5	14	9	3	61
Head and neck cancer	6	2	3	0	1	83
Pancreatic cancer	8	0	4	3	1	50
Previously irradiated	9	3	2	0	4	56
Head and neck cancer	4	1	1	0	2	50
Outside of target volume	9	0	2	3	4	22

CR, complete response; PR, partial response; SD, stable disease; PD, progressive disease; N, patient number.

## Abbreviations:

DC: dendritic cell
RT: radiotherapy
IMRT: intensity-modulated radiotherapy
3DCRT: 3-dimensional conformal radiotherapy
CTL: cytotoxic T lymphocyte
5-FU: 5-fluorouracil
Treg: regulatory T cell
IMAX: immune-maximizing
SBRT: stereotactic body radiotherapy
HLA: human leukocyte antigen
PBMC: peripheral blood mononuclear cell
CR: complete response
PR: partial response
SD: stable disease
PD: progressive disease
MHC: major histocompatibility complex
